# Comparison of seven methods for producing Affymetrix expression scores based on False Discovery Rates in disease profiling data

**DOI:** 10.1186/1471-2105-6-26

**Published:** 2005-02-10

**Authors:** Kerby Shedden, Wei Chen, Rork Kuick, Debashis Ghosh, James Macdonald, Kathleen R Cho, Thomas J Giordano, Stephen B Gruber, Eric R Fearon, Jeremy MG Taylor, Samir Hanash

**Affiliations:** 1 Department of Statistics, University of Michigan, Ann Arbor, Michigan, USA; 2 Department of Biostatistics, University of Michigan, Ann Arbor, Michigan, USA; 3 Department of Pediatrics, University of Michigan, Ann Arbor, Michigan, USA; 4 Comprehensive Cancer Center, University of Michigan, Ann Arbor, Michigan, USA; 5 Department of Pathology, University of Michigan, Ann Arbor, Michigan, USA; 6 Department of Internal Medicine, University of Michigan, Ann Arbor, Michigan, USA

## Abstract

**Background:**

A critical step in processing oligonucleotide microarray data is combining the information in multiple probes to produce a single number that best captures the expression level of a RNA transcript. Several systematic studies comparing multiple methods for array processing have used tightly controlled calibration data sets as the basis for comparison. Here we compare performances for seven processing methods using two data sets originally collected for disease profiling studies. An emphasis is placed on understanding sensitivity for detecting differentially expressed genes in terms of two key statistical determinants: test statistic variability for non-differentially expressed genes, and test statistic size for truly differentially expressed genes.

**Results:**

In the two data sets considered here, up to seven-fold variation across the processing methods was found in the number of genes detected at a given false discovery rate (FDR). The best performing methods called up to 90% of the same genes differentially expressed, had less variable test statistics under randomization, and had a greater number of large test statistics in the experimental data. Poor performance of one method was directly tied to a tendency to produce highly variable test statistic values under randomization. Based on an overall measure of performance, two of the seven methods (Dchip and a trimmed mean approach) are superior in the two data sets considered here. Two other methods (MAS5 and GCRMA-EB) are inferior, while results for the other three methods are mixed.

**Conclusions:**

Choice of processing method has a major impact on differential expression analysis of microarray data. Previously reported performance analyses using tightly controlled calibration data sets are not highly consistent with results reported here using data from human tissue samples. Performance of array processing methods in disease profiling and other realistic biological studies should be given greater consideration when comparing Affymetrix processing methods.

## Background

Affymetrix microarrays are high throughput assays for measuring the expression levels of thousands of gene transcripts simultaneously. This type of microarray measures the expression of each transcript multiple times through a set of "probe pairs". Since the advent of the Affymetrix microarray, numerous methods have been proposed for producing numerical expression summaries for each transcript based on the probe pair data. Several systematic studies have appeared comparing a number of methods on a common basis (e.g. [1-5]). These studies rely heavily on calibration data sets derived from spike-in, dilution series, and mixture experiments for comparing methods. Our goal here was to carry out a comparative study of Affymetrix array processing methods using data sets from typical biological experiments seeking differentially expressed genes in human tissue samples.

The following seven methods are considered here: Dchip [10], GCRMA-EB and GCRMA-MLE [11], MAS5 [12], PDNN [13], RMA [2,3], and TM [[6,7], and ]. While not every popular method is included in our study, several highly distinctive and original approaches are studied. For example, Dchip was one of the first approaches to attempt to learn probe weights directly from the probe data, and RMA pioneered the approach of disregarding the control mismatch probes. PDNN uses physical modeling to determine probe weights, while the two GCRMA methods use GC content of the probe sequences to reduce variance in the mismatch (control) probe levels. The MAS5 method is the current default method provided by Affymetrix.

In addition to the six methods cited previously, we also include a method designated TM (trimmed mean). This is a simple method that has been used in a number of published investigations (e.g. [6,7]), but has not been considered in any previous systematic comparison of Affymetrix processing methods. To produce the probe-set summary score, the PM-MM differences are rank ordered, and the brightest 20% and dimmest 20% of values are deleted. The mean of the remaining values is used as the summary score. The scores for all probe-sets are then quantile normalized to a reference array using a piecewise linear spline with 100 knots.

An important feature of this study is the use of False Discovery Rate (FDR) to quantify the sensitivity of a processing method in terms of its ability to distinguish differentially expressed genes from genes having invariant expression. This is a highly relevant property, as differential expression analysis is the most common application of microarray data. A key advantage of using FDR to compare processing methods is that FDR values can be calculated accurately using real disease profiling data where the identities of differentially expressed genes are uncertain. In contrast, most previous systematic comparisons of array processing methods have focused on calibration data sets in which concentrations of certain genes were experimentally manipulated.

When it is highly likely that at least one gene is differentially expressed, false discovery rate may be defined as the expected ratio of the number of false positive calls to the total number of positive calls in a differential expression analysis between two groups of samples [8]. If the groups are biologically distinct, a sensitive processing method should result in many genes with low FDR. Thus to compare the performances of different array processing methods, we looked at two datasets in which a verified biological characteristic divided the samples into two classes, and compared the methods based on the number of genes having FDR smaller than various thresholds. For this to be a valid basis for comparison, the FDR values must be estimated with reasonable accuracy. Following other recent work (e.g. [9]), we used a permutation approach for this estimation, arguing that there is no reason that this approach favors or disfavors any particular array processing method.

A small FDR is due either to a small numerator, a large denominator, or both. The denominator of the FDR depends on the actual data distribution, so variation in this value may be due to factors such as accuracy in modeling the physical and chemical nature of probe binding. Variation in the FDR numerator, however, depends only on the distribution of values produced for randomized data, a purely statistical quantity reflecting the tendency of the method to incorrectly produce test statistic outliers. Our results suggest that both factors are important in determining sensitivity. The best methods produce many large test statistic values in the actual data, and also produce consistently small test statistic values for randomized data. Poor performance of one method can be directly explained by the tendency of the method to produce outlier expression values, leading to greater numbers of incorrectly large test statistics.

For overall comparison, we evaluated every pair of methods on the basis of whether the first method is expected to call at least one truly differentially expressed gene that is not also called by the second method. If this is not expected to occur, the second method is said to *strongly outperform *the first. Based on this comparison, two of the methods considered are clearly favored, two are inferior, and results for the other three methods are mixed.

## Results

### Sensitivity differences

**Figure 1:**
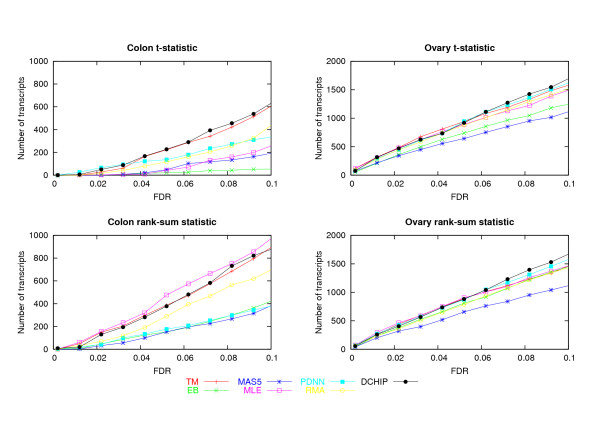
**Sensitivity results for colon and ovary data. **Top row: number of significant probe sets at a range of FDR_0 _values using the t-test statistic. Bottom row: number of significant probe sets at a range of FDR_0 _values using the rank-sum statistic. The left column shows the results for colon data and the right column shows the results for ovary data.

Our primary basis for comparison is sensitivity – the number of genes detected at a given FDR_0 _level, where FDR_0 _is a rescaled FDR (see methods). Figure 1 shows the key sensitivity results, using both the t-test statistic and the rank-sum statistic to assess differential expression. Setting aside at first differences between the seven processing methods, we note two findings. First, in the colon data, analysis using the rank-sum statistic is substantially more sensitive than analysis using the t-test statistic. For the ovary data, where the sample sizes would not naturally suggest a robust analysis, there is no harm to sensitivity in using the rank-sum statistic. Second, the ovary curves are substantially higher overall than the colon curves. This may be due to a greater number of true positives in the ovary data, or it may be that the small sample size for the MSI group makes it difficult to attain high evidence levels for differential expression in the colon data. In any case, both data sets have many genes with small FDR values, supporting the biological relevance of the tumor groupings for both colon and ovary samples.

The more challenging colon set distinguishes the seven processing methods to a greater extent than the ovary set. Using FDR_0 _= 0.1 as a reference point, there is roughly 7-fold variation across the seven methods in the number of detected genes in colon data using t-test statistics, while for rank-sum statistics the range is roughly 2-fold. In the ovary data, the range is around 1.25-fold for both statistics. Also notable is that variation in sensitivity due to the choice of test statistic (t-test or rank-sum) is smaller than variation in sensitivity due to the seven processing methods.

No single method stands out as having the best or worst performance in every case. However some methods generally perform better than others. The Dchip and TM methods perform consistently well, while the GCRMA-EB and MAS5 methods consistently perform poorly. PDNN performs well on the ovary data, but poorly on the colon data, and results for the other methods are mixed.

### Level of agreement between methods

**Figure 2:**
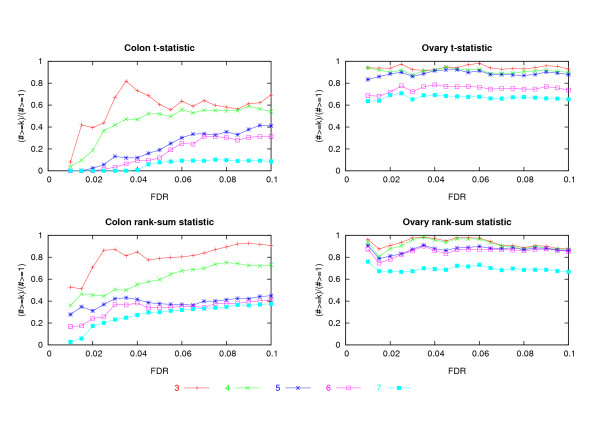
**FDR agreement between methods. **The ratio of the number of probe sets with FDR_0 _value below a given threshold in *k *or more of the seven methods to the number of probe sets with FDR_0 _value below the threshold in at least one method was calculated for *k *= 3, 4, 5, 6, 7, and plotted against the FDR_0 _threshold. Results are shown for the colon data (left column), the ovary data (right column), and for the t-test statistic (top row), and the rank-sum statistic (bottom row).

Identities of probe sets falling below a given FDR_0 _threshold vary across the methods. Figure 2 summarizes this variation. The ratio of the number of probe sets falling below various FDR_0 _thresholds in *k *or more of the seven methods to the number of probe sets falling below the threshold for at least one method is plotted against the FDR_0 _threshold, for *k *= 3, 4, 5, 6, 7. In the ovary data there is a very high level of agreement in this measure. For the rank-sum analysis, almost 90% of called genes are called by at least four methods, and more than 70% of called genes are called by all seven methods. For the t-test analysis, the agreement is slightly higher yet. For the colon data, the methods are much more inconsistent. For the rank-sum analysis, three of the methods agree on up to 90% of genes, but all seven methods only agree on around 30% of genes. The t-test analysis is even worse, with only around 10% of genes common to all seven methods.

Turning to pairwise agreement, Table 1 shows the percentage of genes called by both members of a pair of methods out of the genes called by at least one of the two methods. In the ovary data, MAS5 shares the fewest calls with the other methods for both t-test and rank-sum analysis, while GCRMA-EB has relatively weak agreement for the t-test analysis. In the colon data, the GCRMA-EB method is highly inconsistent, with less than a quarter of calls in common with four of the six other methods for t-tests. A notable similarity is that the DChip and TM methods have at least 90% agreement in all analyses.

Complementing comparison of the statistical tests, we also compared the expression levels produced by the seven processing methods. For each pair of methods, and for each pair of samples within one of the two data sets, we calculated Pearson correlation coefficients of expression levels over all genes. These values were summarized by taking the median over all pairs of samples within a data set, shown in Table 2. Interestingly, methods calling similar genes as differentially expressed do not exhibit particularly strong correlation in expression levels. For example, TM and DChip perform very similarly in terms of which genes are identified as significant, but the pairwise correlation between expression levels for these two methods is less than the average. On the other hand, the TM and MAS5 methods are generally at the extreme high and low ends of the sensitivity scale respectively, but their expression levels are the most strongly correlated of any pair of methods.

### Calibration

Variation in FDR across the seven methods is due to two factors – variation in the number of transcripts with large test statistics, and variation in the expected number of transcripts with large test statistics when there is no real differential expression. Here we investigate the second factor, which is driven by the tendency of each method to produce outlier expression values.

The numerator of the FDR aims to correct for variation in the number of false positives, so that a method claiming large numbers of differentially expressed genes is not considered superior unless it also produces relatively small numbers of false positives. This can be viewed as a calibration, in which for each method, the test statistic must reach a certain threshold in order that the proportion of false positives is no greater than a specified value.

**Figure 3:**
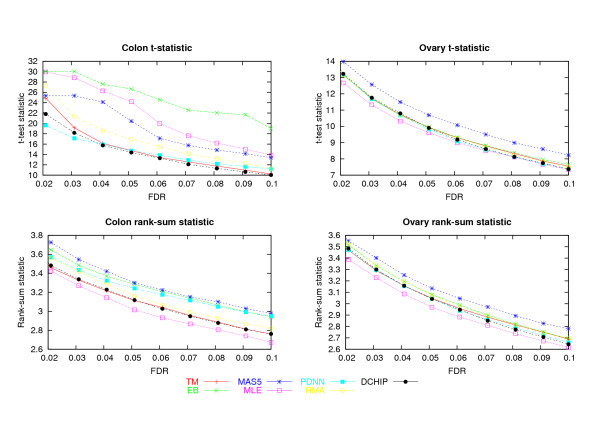
**Calibration results for ovary and colon data. **The threshold test statistic required to obtain a given FDR_0 _for each method is plotted against the FDR_0 _value. Results are shown for the colon data (left column), the ovary data (right column), and for the t-test statistic (top row), and the rank-sum statistic (bottom row).

Calibration results are summarized in Figure 3. For each method, the threshold test statistic value required to obtain FDR_0 _less than *f *was calculated, and plotted against *f*. For example, to achieve any FDR_0 _value between 0.05 and 0.1 in the colon rank-sum data, GCRMA-MLE requires the lowest test statistics, RMA requires a rank-sum statistic 0.15 units larger than that of GCRMA-MLE, and MAS5 requires a rank-sum statistic 0.3 units larger than that of GCRMA-MLE.

Figure 3 indicates that the methods differ substantially in terms of calibration. Notably, the ordering of the seven methods in Figures 1 and 3 are quite similar, suggesting that calibration plays a major role in determining sensitivity. Variation in thresholds among the seven processing methods is greater in the colon than the ovary data, particularly for the t-test analysis.

Since calibration depends only on randomized data, it should be possible to trace variation in thresholds across the processing methods to statistical properties of the expression levels. For example, if one method produces expression levels with heavier tails, it is easier to get a large t-test statistic value by chance, particularly for the colon data with small sample sizes. This would necessitate a higher threshold. To quantify this, let 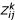 denote the log_2 _expression level of transcript *i *in sample *j *for method *k*, where *k *= 1, ..., 7 denotes the seven processing methods, and let


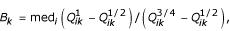


where 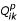 is the *p*^th ^quantile of 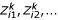, and med is the median value. This is an affine-invariant measure of the size of the right tail of the expression values. Values of *B*_*k *_for the seven methods and two data sets are shown in Table 3. For reference, a Gaussian distribution has a *B *value of 3.74 when the sample size is as in the ovary data, and 3.56 when the sample size is as in the colon data. The GCRMA-EB method is seen to have a much greater propensity for producing extreme expression values, explaining its low sensitivity, poor agreement with other methods, and conservative calibration.

### Variation in observed test statistics

**Figure 4:**
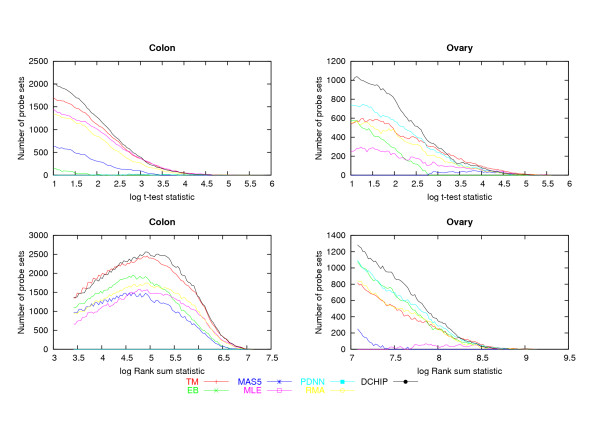
**Test statistics for ovary and colon data. **For each of the seven processing methods, the number of probe sets exceeding a test statistic threshold *t *was calculated and plotted against log_2 _t. Results are shown for the colon data (left column), the ovary data (right column), and for the t-test statistic (top row), and the rank-sum statistic (bottom row).

In addition to calibration differences, FDR variation is also influenced by the observed test statistic values. This is summarized in Figure 4. For each method, and for a range of test statistic values *t*, the number of probe sets for which the observed test statistic value exceeds *t *was calculated and plotted against *t*. For example, in the colon rank-sum data, PDNN had the smallest test statistics, with MAS5 having around 500 more probe sets meeting a log test statistic threshold of 5 compared to PDNN. The Dchip and TM methods have over a thousand more probe sets meeting this threshold.

Variation in test statistic values across the methods is greater in colon than in ovary data, and generally tracks with sensitivity. However note that in the colon rank-sum data, Dchip has substantially larger test statistics than GCRMA-MLE, even while GCRMA-MLE has better sensitivity (Figure 1), due to its less stringent calibration (Figure 3).

### Identification of genes with large fold changes

An interesting possibility that can not always be excluded is that the intergroup differences are so vast that nearly every gene is affected to a small degree. If this were the case, the FDR values for the t-test and Wilcoxon statistics would converge to zero for every gene as the number of samples grows, making FDR values difficult to interpret. To further investigate this issue, we repeated the analysis using t-statistics truncated to zero when the fold change is less than 1.5 as test statistics for FDR analysis. The corresponding FDR values remain bounded away from zero for genes having true fold change smaller than 1.5, while genes with true fold change exceeding 1.5 have FDR values converging to 0. Thus the statistic identifies a meaningful subset of genes even when all genes are differentially expressed to some degree.

**Figure 5:**
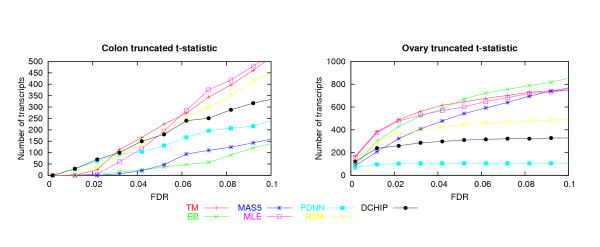
**Sensitivity for detecting genes with at least 50% change in expression magnitude. **The number of significant probe sets at a range of FDR_0 _values is shown for analysis in which the test statistic is the t-statistic truncated to zero when the fold change is less than 50%.

Results for this analysis are shown in Figure 5. In the ovary data, the GCRMA-EB method performs best, with GCRMA-MLE, MAS5, and TM slightly inferior. Several of the methods, specifically PDNN, DChip, and RMA exhibit flat curves indicating that only a limited number of genes meet the 50% change criterion. In the colon data, GCRMA-MLE and TM are nearly tied as the best performers. Overall, variation in sensitivity across the methods exists at a similar level to that found in the t-test and Wilcoxon analyses. Only the GCRMA-MLE and TM methods give consistently good performances in the two data sets for this analysis.

### Strong outperformance

Thus far we have focused on sensitivity as a criterion for comparing methods. However even if one method is less sensitive than another, if the overlap in the called gene sets is not too great then the less sensitive method may still contribute to our understanding of which genes are differentially expressed. Suppose two methods denoted 1 and 2 give *N*_1 _and *N*_2 _genes respectively at a given FDR level. Then *n*_*k *_= (1 - *p*_0_FDR_0_)·*N*_*k *_estimates the expected number of truly differentially expressed genes called by method *k*. Now suppose that *I *is the number of genes called by both methods. Then *n*_*k *_- *I *is an estimated lower bound for the expected number of genes correctly called by method *k *but not by the other method. We will say that method 1 *strongly outperforms *method 2 if *n*_1 _- *I *≥ 0 but *n*_2 _- *I *< 0. This means that in terms of differential expression, method 2 is not expected to contribute any true positives that were not called by method 1.

Table 4 summarizes the results of this analysis using *p*_0 _= 1 and FDR_0 _= 0.05, showing the number of times that each method was strongly outperformed by other methods in our study. This analysis clearly favors the TM and Dchip methods, while the MAS5 and GCRMA-EB methods are nearly always found to be strongly outperformed by the other 5 methods. These results are not sensitive to choices of *p*_0 _between 0.5 and 1 (more than half of values are constant within this range and non-constant values do not vary by more than 1).

## Discussion

### Impact of processing method choice

The choice of processing method for Affymetrix array data evidently has a major impact on the ability to confidently report the results of differential expression analysis. The effect is greater, for example, than the choice of using a robust or a non-robust analysis, even in the colon data where robust analysis results in substantial improvements. Differences among processing methods are much greater in the more challenging colon data set compared to the ovary data, yet it should be noted that the sample sizes in the colon data are not atypical in real investigations.

While results from two data sets can never conclusively determine the optimal method, it is notable that across both data sets, using both t-statistic and rank-sum analyses, there is a high degree of similarity in the rank ordering of the methods from the best to the worst performer. The trimmed mean (TM) and Dchip methods consistently perform as well or better than any of the other methods. A possible explanation for this is that the weights used by the Dchip may tend to downweight the least and greatest PM-MM differences, just as the TM method excludes these differences.

### Interpretation of FDR comparisons

When comparing array processing methods using experimental data in which the identities of differentially expressed genes are unknown, great care must be taken to ensure that apparent differences in sensitivity are not due to other factors. One critical point is that the null distribution providing the expected number of false positives at a given test statistic threshold (the numerator of the FDR) must fairly reflect the statistical behavior of null genes. Permutation approaches have been extensively used to produce empirical p-values (e.g. [14]) and were used by Efron et al. [9] to estimate FDR values. Although permutation approaches are known to be slightly biased for estimating the FDR, the size of the bias (e.g. as shown in figure 5 of Efron et al. [9]) can not explain the magnitude of differences found here. In addition, for a comparative analysis, as carried out here, it is more crucial that the biases be relatively constant across the methods. However, since permutation approaches may not be highly accurate when the sample size is small, it is important to check performance on multiple data sets before conclusions about performance are drawn.

While we have focused on FDR as the basis of comparison, the pursuit of small FDR values is not the only desirable operating characteristic of an array processing method, and other reports have also emphasized the accuracy of estimating the precise size of concentration differences. However to the extent that most actual studies seek to find differential expression between groups, the use of small FDR values seems more instrumental as the basis for judging methods.

### Variation due to choice of test statistic

Although our primary aim was to investigate variation in sensitivity due to the seven processing methods, all analysis was carried out independently for two test statistics. The t-statistic is widely used in practice, but is well-known to be sensitive to outliers, particularly when the sample size is small. We found that certain processing methods, particularly EB-GCRMA, had a tendency to produce outlier expression values in the colon data set. Thus the combination of using the EB-GCRMA method with t-statistics in the colon data led to particularly poor performance.

### Variation due to log transform and array normalization

In practice, the approach used for array normalization and for forming log-transformed expression values may be equally or more influential than the method used for producing probe set summaries [15]. In this study, we used implementations of the seven processing methods as prepared by their developers, and thus array normalization and and log-transforms were applied in a method-specific fashion. This provides a comparative analysis of the various methods as they are used in practice, which is most directly relevant since few investigators will override the default normalization and log-transform methods provided by the developers of each method.

Nevertheless it remains of interest whether these routine processing steps are the determining factor of performance. In a future study it will be important to investigate this question further by modifying the implementations of the processing methods so that uniform log transforms and array normalizations are applied.

### Comparison of methods using data from disease profiling data sets

A key point that we advocate in this work is that false discovery rates in actual disease profiling data constitute a valuable complement to benchmarking results obtained from spike-in, dilution series, and mixture experiments (e.g. [4,5]). The primary obstacle that must be overcome is that proper null sampling distributions are essential to ensure that the methods are compared on a common basis. Since numerous data sets covering a wide range of Affymetrix platforms are available, to the extent that multiple data sets are in agreement about relative performances it is unlikely that the randomization procedure used to calculate FDR values is systematically biased against a particular method.

In spite of the statistical challenges in using disease profiling data for benchmarking, we argue that these data sets also offer some unique advantages. Calibration data sets are relatively few in number and are not available for all platforms. Newer platforms in particular are under-represented. Therefore overtraining to the available calibration data through manipulation of the many tuning parameters in the more complicated processing methods is an unavoidable concern. In addition, the calibration data sets likely do not represent the same degree of challenge as disease profiling data in that reproducibility of fold changes for affected and unaffected genes is quite high compared to data from, say, human tissues where a large number of uncontrolled sources of variability are present.

## Conclusions

Performances of multiple array processing methods on disease profiling data sets vary widely across the seven methods studied here, but results are generally consistent between the two data sets studied. Results of our analysis generally do not parallel results obtained using calibration data sets [4,5], suggesting that such comparisons may not completely capture the most relevant aspects of performance.

A major determinant of sensitivity is test statistic variability for randomized data. Such variability will affect false discovery rates as well as empirical p-values, which are an often-used alternative approach for identifying differentially expressed genes (e.g. [14]). Therefore it will be important in future work to seek a better understanding of statistical sampling properties of array processing methods. A particular focus should be the way that sampling variance in probe masking and probe weighting is controlled. Methods seeking to incorporate mechanistic information about the dynamics of probe binding, such as the two GCRMA methods and PDNN, should in principal outperform more generic approaches such as the TM method. Our results, particularly in the colon data, suggest that in medium-sized data sets this potential is not yet reached.

In this comparative analysis we did not seek to draw definitive conclusions about the "best" or "worst" methods. Such conclusions may be made after investigating a greater number of data sets, including disease profiling data, data from controlled experiments, and calibration data. Moreover, it may be that the correct choice of method may depend on the scientific question being asked. The key message of this work is that the wide range of data sets collected in actual scientific investigations may be used for comparison of processing methods, and that in at least the two data sets considered here, similar results were obtained in the rank ordering of the methods.

## Methods

### Data sets

We used two data sets – one consisting of 79 ovary tumors and the other consisting of 47 colon tumors. Both sets were generated at the University of Michigan using Affymetrix HG_U133A arrays, which consist of 22283 probe-sets, each of which is designed to assay a RNA transcript. Each probe-set consists of a set of (typically 11) probe-pairs, with each probe-pair comprising a "perfect match" (PM) probe which is a 25-base oligonucleotide complementary to the transcript, and a "mismatch" (MM) probe that is identical to the PM sequence except for alteration of the central base. The MM probe is intended as a control for nonspecific hybridization, so that the difference PM-MM measures only specific binding. However not all processing methods use the MM data in this way.

For differential expression analysis, the 79 ovary samples were partitioned according to histological class into 38 endometrioid and 41 serous samples. The 47 colon samples were partitioned into 40 microsatellite stable (MSS) samples and 7 microsatellite instable samples (MSI). In both data sets, the partition is based on an independently measured biological characteristic, so there almost certainly are differentially expressed genes to be found. However in neither case are the two classes highly distinct, and numerous other sources of biological variation are undoubtedly present in the data.

### Normalization across arrays

Array normalization refers to an adjustment of data distributions within each array in order to make the arrays more comparable. Each array processing method has been coupled with a normalization procedure by its developers (see references). We followed these method-specific normalization practices in our analysis. All methods other than MAS5 use some form of quantile normalization.

### Log transform and truncation

All analysis was based on log-transformed data. Log-transformed values, including truncations where needed, were calculated in the manner recommended by the developers of each method (see references).

### Methodology of comparison

We compared the seven methods based on their sensitivity in detecting differential expression at a fixed false discovery rate (FDR). For each method, two different two-sample test statistics were calculated for each gene – the standard two-sample t-statistic, and the Wilcoxon rank-sum statistic (equivalent to the Mann-Whitney statistic). The t-test statistic *T *is always analyzed as |*T*|, and the rank-sum statistic *R *is standardized as 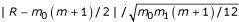, where *m*_0_, *m*_1 _are the numbers of samples in the two classes, and *m *= *m*_0 _+ *m*_1 _is the total number of samples.

Our FDR approach closely follows the "global estimate" of Efron et al. ([9] equation 5.9). For a given test statistic threshold *t*, the FDR was estimated as follows. Randomized data sets were constructed by randomly reassigning the class identifiers to the samples. The average number of transcripts with test statistic value exceeding *t *was calculated over 1000 randomized data sets. This number was divided by the number of actual transcripts with test statistic value exceeding *t *to produce a value that we denote FDR_0_. In practice the value of FDR_0 _should be scaled by the proportion *p*_0 _of non-differentially expressed genes, giving FDR = *p*_0_FDR_0_. Although various estimates of *p*_0 _exist, we elected to ignore this factor since it is constant across the methods for a given data set, and any estimate of *p*_0 _would add an additional source of uncertainty to our results. Thus it should be noted that the reported FDR_0 _values, while comparable across methods, are somewhat larger than the usual estimates. Since *p*_0 _would generally be greater than 1/2, the bias is likely less than a factor of 2.

## Authors' contributions

KS, JMGT, RK, and DG participated in all phases of design and analysis. WC and JM performed the data analysis. KRC, TJG, SBG, ERF, and SH assisted in study design and supervised data collection. All authors read and approved the final manuscript.

## Figures and Tables

**Table 1:** Pairwise agreement between methods. For each pair among the seven processing methods, the ratio of the number of probe sets with FDR_0 _< 0.05 in both methods to the number of probe sets with FDR_0 _< 0.05 in either method was calculated. Results are displayed as percentages.

		**Colon**						**Ovary**					
**t-test**	EB	9						77					
	MAS5	19	45					68	88				
	MLE	15	60	60				94	81	72			
	PDNN	68	14	28	23			93	83	73	98		
	RMA	58	16	32	26	86		92	83	74	98	100	
	DCHIP	90	8	17	14	60	52	95	81	71	99	97	97
	
**Rank-Sum**	EB	39						90					
	MAS5	38	88					73	82				
	MLE	84	32	32				99	89	72			
	PDNN	45	86	84	38			98	91	74	97		
	RMA	71	54	53	60	63		88	98	83	87	89	
	DCHIP	94	41	40	79	48	75	99	91	74	98	99	89
	
		TM	EB	MAS5	MLE	PDNN	RMA	TM	EB	MAS5	MLE	PDNN	RMA

**Table 2:** Median pairwise correlations over all sample pairs. For each pair of processing methods, expression levels were computed for each sample in the colon and ovary data sets. Results shown are the median Pearson correlation coefficients over all sample pairs between log-scale expression levels for all genes.

	**Colon**						**Ovary**					
GCRMA-EB	0.86						0.86					
MAS5	0.94	0.84					0.95	0.84				
GCRMA-MLE	0.88	0.80	0.91				0.89	0.80	0.92			
PDNN	0.82	0.77	0.74	0.71			0.82	0.78	0.76	0.73		
RMA	0.88	0.81	0.85	0.82	0.85		0.89	0.80	0.86	0.85	0.85	
DChip	0.81	0.73	0.76	0.75	0.81	0.89	0.83	0.74	0.79	0.77	0.83	0.90
	TM	GCRMA-EB	MAS5	GCRMA-MLE	PDNN	RMA	TM	GCRMA-EB	MAS5	GCRMA-MLE	PDNN	RMA

**Table 3:** Tendencies of the processing methods to produce outlier expression values. Values of the *B *statistic (see text) are shown for the seven processing methods and two data sets.

	Colon	Ovary
TM	3.71	4.03
GCRMA-EB	6.75	6.21
MAS5	3.44	3.74
GCRMA-MLE	5.09	5.43
PDNN	3.49	4.84
RMA	4.34	4.90
Dchip	3.82	4.39

**Table 4:** Strong outperformance of each method. For each of the seven processing methods, and for each of the four analyses, the number out of the other 6 processing methods that strongly outperform the given method at FDR = 0.05 was determined.

	Colon		Ovary	
	t	RS	t	RS
TM	1	1	0	0
GCRMA-EB	5	5	5	4
MAS5	4	5	6	6
GCRMA-MLE	4	0	1	0
PDNN	2	4	1	0
RMA	3	3	1	4
DChip	0	2	0	0
